# First person – Nagisa Yoshida

**DOI:** 10.1242/dmm.045922

**Published:** 2020-07-20

## Abstract

First Person is a series of interviews with the first authors of a selection of papers published in Disease Models & Mechanisms, helping early-career researchers promote themselves alongside their papers. Nagisa Yoshida is first author on ‘The zebrafish as a novel model for the *in vivo* study of *Toxoplasma gondii* replication and interaction with macrophages’, published in DMM. Nagisa conducted the research described in this article while a PhD student in Prof. Serge Mostowy and Dr Eva Frickel's labs at Imperial College London/The Francis Crick Institute/London School of Hygiene and Tropical Medicine, London, UK. She is now a postdoc in the lab of Dr Robindra Basu-Roy and Prof. Serge Mostowy at London School of Hygiene and Tropical Medicine, and her research interests include infection biology and immunology.


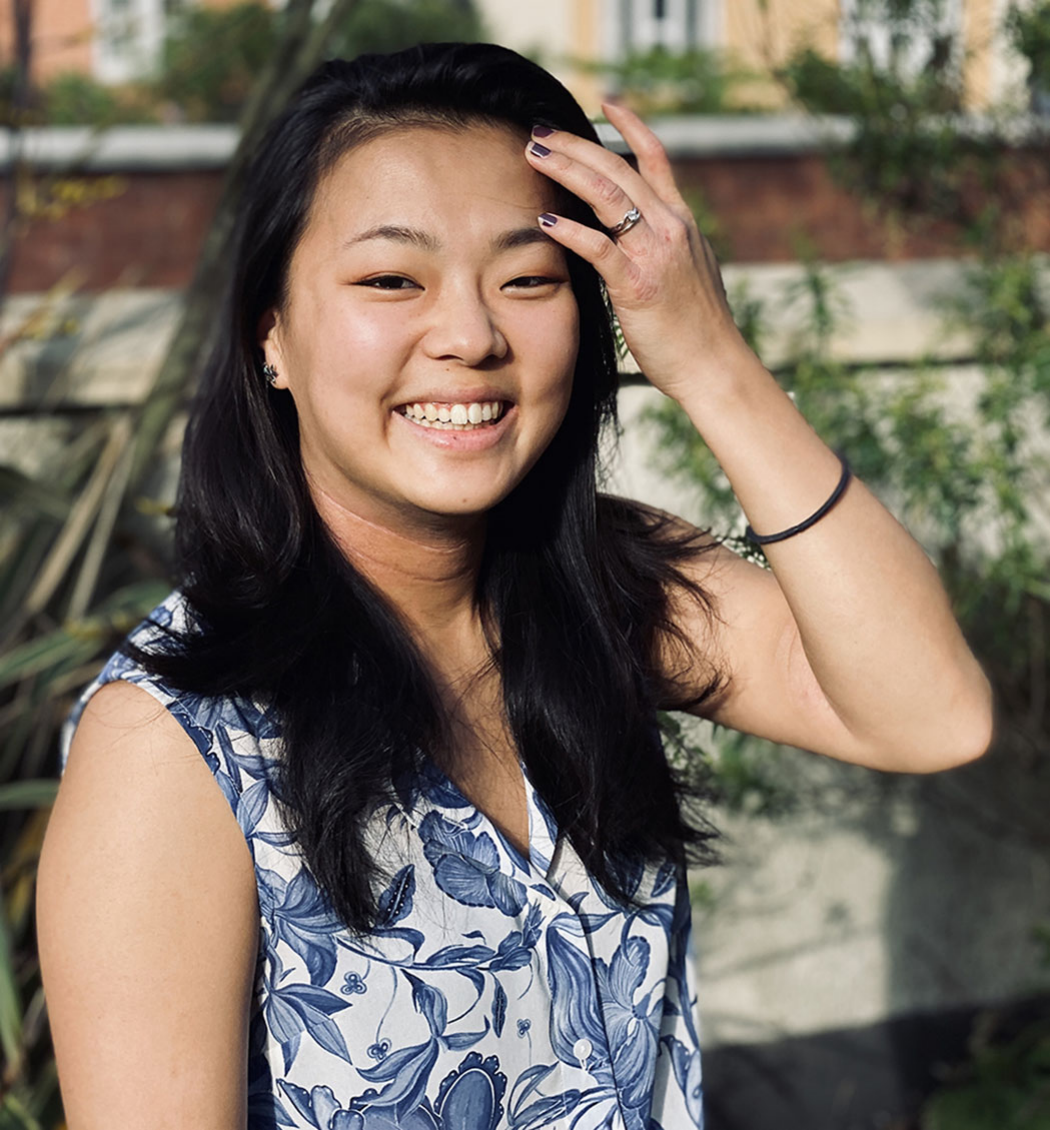


**Nagisa Yoshida**

**How would you explain the main findings of your paper to non-scientific family and friends?**

The parasite *Toxoplasma gondii* is commonly associated with the term ‘crazy cat-lady syndrome’, which may not be far from the truth. Long-term (chronic) *Toxoplasma* infection has been linked to changes in behavior, including a reduced fear response to the smell of cat urine in mice, which is important because the primary host *Toxoplasma* infects is the cat, a natural predator of the mouse. With one in three people on average infected with *Toxoplasma* without any outward symptoms, and no treatment that can totally eradicate the parasite from the host, understanding how *Toxoplasma* survives and interacts with the immune system is critical. The difficulty arises when we want to see the parasite in action inside a living organism. To see anything happening inside a mouse requires specialized skills and equipment and can be invasive – this is where the zebrafish comes in. While many may think, “zebrafish can't be similar to humans”, genetic studies have shown that ∼70% of the genetic blueprint of zebrafish (i.e. their DNA) is similar to that of humans, and when we look specifically at genes that cause disease in humans this similarity goes up to ∼82%. A big advantage of using zebrafish larvae (less than 5 days old) is that during this time larvae are translucent, which means that all imaging happens non-invasively and only requires the zebrafish to be anesthetized. Using state-of-the-art microscopes to visualize zebrafish (modified to have fluorescent immune cells) infected with fluorescent *Toxoplasma*, we have shown that macrophages (first responders of the immune system that eat and digest foreign objects) predominantly clear *Toxoplasma* in zebrafish, and that macrophages could also be invaded by *Toxoplasma* to help the parasite grow or move around the brain tissue. Visualizing *Toxoplasma*-macrophage interactions has been very difficult up until now in other animal models. Every cell in our body possesses weapons that can directly attack and break the membrane that surrounds and protects the parasite (i.e. the parasitophorous vacuole). Previous work has only shown this in mouse and human cells cultured outside of the body. Here, our data have shown that this happens in macrophages in zebrafish, and we also found broken parasite membranes in brain cells, something that was previously only speculated.

**What are the potential implications of these results for your field of research?**

When studying a parasite that is so intricately involved with the central nervous system, highly specialized microscopes are needed to study host-parasite interactions in real time. By developing a *Toxoplasma*-zebrafish infection model, we have established a new model that can evolve and adapt to the question being asked. Here, we describe the immediate innate immune response to *Toxoplasma* infection (i.e. the non-specific defense system of the body), focusing on macrophages in the context of brain tissue of larvae <5 days old. After understanding these foundations, our model can be extrapolated to study (for example) precise innate immune mechanisms using knockout zebrafish lines, chronic infection, the impact of *Toxoplasma* infection on neuron function and behavior in a whole organism, and whether macrophages can act as a ‘Trojan horse’ that aids parasite dissemination into immune privileged sites. The model we describe here is just the starting point.

**What are the main advantages and drawbacks of the model system you have used as it relates to the disease you are investigating?**

An advantage of injecting into the zebrafish hindbrain ventricle is that it allows for easy visualization of leukocyte recruitment, but more importantly allows us to directly investigate *Toxoplasma* infection of brain tissue in the context of a whole organism, something that is difficult to visualize in mouse models of infection. However, there are still many unknowns in the zebrafish genome, even though zebrafish have counterparts to ∼70% of the human genome. Lessons learned using the zebrafish model may not necessarily translate directly to the human system, and so findings must be tested in higher vertebrates before discoveries can be extrapolated to the human system.

“Our research has provided a first look at what neurons are capable of in response to *Toxoplasma* infection.”

**What has surprised you the most while conducting your research?**

I had two major surprises during my PhD research. First, the complete lack of zebrafish mortality observed as a result of *Toxoplasma* infection. Looking back, the mortality should not have been that much of a surprise considering that *Toxoplasma* is mostly harmless in healthy individuals. Second was the realization that our electron microscopy data (obtained with help from Marie-Charlotte Domart and Christopher Peddie at The Francis Crick Institute) showed that brain cells, as well as macrophages, could break the vacuole around the parasite. Electron microscopy is a technique used for obtaining high-resolution detailed images of structures inside a cell that you would not be able to see with a normal microscope, let alone the naked eye. It was striking to be able to see *Toxoplasma* inside a cell in a whole organism at that level of detail. Breakage of the vacuole around the parasite by host cells had previously been observed in both human and mouse cells. Whether neurons also have the ability to directly kill *Toxoplasma* tachyzoites in an organism has been unclear until now. Our research has provided a first look at what neurons are capable of in response to *Toxoplasma* infection.

**Describe what you think is the most significant challenge impacting your research at this time and how will this be addressed over the next 10 years?**

The most significant challenge (apart from the COVID-19 pandemic) is automation in zebrafish infection research. In particular, image analysis requires a lot of manual work and hours of dedication. Removing this from the equation could eliminate any unconscious bias and free up hours of a researcher's time that is currently being taken up sitting in front of a computer and manually counting parasites. There are great new advances out there that have started to address these challenges (including automated quantifications we have performed in this paper with the help of ZedMate, developed by Artur) but the field is continually progressing and there is still a way to go before this becomes routine.

**Figure:**
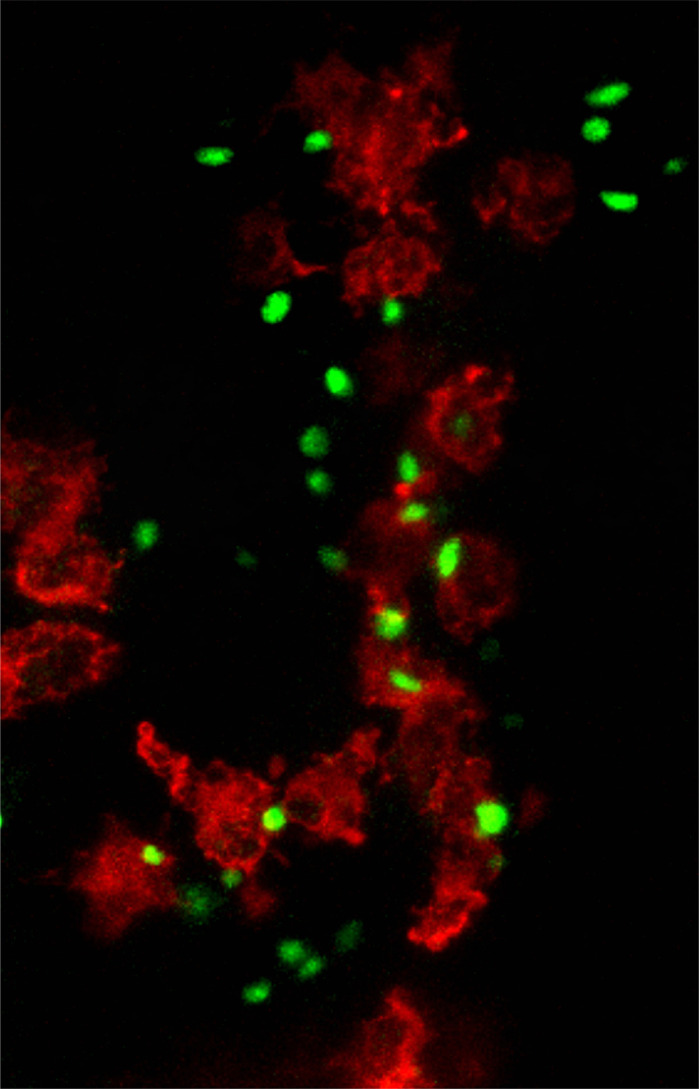
**Confocal image of the hindbrain of 3-day-old *mpeg1*:*G/U*:mCherry zebrafish larvae harboring red macrophages infected with *Toxoplasma gondii* (green) at 6 h post-infection.**

“With guidance and encouragement to step out of their comfort zone, the next generation of scientists can more easily find their place in this increasingly competitive landscape.”

**What changes do you think could improve the professional lives of early-career scientists?**

From my experience, mentorship and perspective. There is a lot of pressure as a PhD student to produce results and write reports. While it is clearly necessary, it is important as a mentor to actively encourage students to take the time to step back (breathe), not only to think about their current research but to also explore the career avenues that are open to them. It is easy to become so involved with the research that we forget that we are not yet fully fledged scientists. With guidance and encouragement to step out of their comfort zone, the next generation of scientists can more easily find their place in this increasingly competitive landscape.

**What's next for you?**

Although currently interrupted due to COVID-19, I am working with Dr Basu-Roy, a clinician scientist using the *Mycobacterium marinum*-zebrafish infection model to explore novel therapeutic strategies to improve the killing of bacteria and disease outcome. In the future, I am looking to further broaden the skillsets I have developed during my PhD so I can contribute to advancing our understanding of infection biology and immunity.
